# IL7 genetic variation and toxicity to immune checkpoint blockade in patients with melanoma

**DOI:** 10.1038/s41591-022-02095-5

**Published:** 2022-12-16

**Authors:** Chelsea A. Taylor, Robert A. Watson, Orion Tong, Weiyu Ye, Isar Nassiri, James J. Gilchrist, Alba Verge de los Aires, Piyush Kumar Sharma, Surya Koturan, Rosalin A. Cooper, Victoria K. Woodcock, Elsita Jungkurth, Brian Shine, Nicholas Coupe, Miranda J. Payne, David N. Church, Vivek Naranbhai, Stefan Groha, Paul Emery, Kulveer Mankia, Matthew L. Freedman, Toni K. Choueiri, Mark R. Middleton, Alexander Gusev, Benjamin P. Fairfax

**Affiliations:** 1 MRC Weatherall Institute of Molecular Medicine, University of Oxford, Oxford, UK; 2 Department of Oncology, University of Oxford, Oxford, UK; 3 Oxford Cancer and Haematology Centre, Oxford University Hospitals NHS Foundation Trust, Churchill Hospital, Oxford, UK; 4 Department of Paediatrics, University of Oxford, Oxford, UK; 5 Wellcome Centre for Human Genetics, University of Oxford, Oxford, UK; 6 Department of Clinical Biochemistry, Oxford University Hospitals NHS Foundation Trust, John Radcliffe Hospital, Oxford, UK; 7 Department of Medical Oncology, Dana-Farber Cancer Institute, Harvard Medical School, Boston, MA USA; 8 Massachusetts General Hospital Cancer Center, Boston, MA USA; 9 Center for the AIDS Programme of Research in South Africa, Durban, South Africa; 10 Department of Medical Oncology, Division of Population Sciences, Dana-Farber Cancer Institute, Boston, MA USA; 11 Broad Institute of Harvard & MIT, Cambridge, MA USA; 12 Harvard Medical School, Boston, MA USA; 13 Leeds Institute of Rheumatic and Musculoskeletal Medicine, University of Leeds, Leeds, UK; 14 National Institute for Health Research Leeds Biomedical Research Centre, Leeds Teaching Hospitals NHS Trust, Leeds, UK; 15 Lank Center for Genitourinary Oncology, Dana-Farber Cancer Institute, Boston, MA USA; 16 Department of Medicine, Brigham and Women’s Hospital, Boston, MA USA; 17 NIHR Oxford Biomedical Research Centre, Oxford University Hospitals NHS Foundation Trust, Oxford, UK; 18 Division of Genetics, Brigham and Women’s Hospital, Boston, MA USA

**Keywords:** Genetics research, Melanoma

## Abstract

Treatment with immune checkpoint blockade (ICB) frequently triggers immune-related adverse events (irAEs), causing considerable morbidity. In 214 patients receiving ICB for melanoma, we observed increased severe irAE risk in minor allele carriers of rs16906115, intronic to *IL7*. We found that rs16906115 forms a B cell-specific expression quantitative trait locus (eQTL) to *IL7* in patients. Patients carrying the risk allele demonstrate increased pre-treatment B cell *IL7* expression, which independently associates with irAE risk, divergent immunoglobulin expression and more B cell receptor mutations. Consistent with the role of IL-7 in T cell development, risk allele carriers have distinct ICB-induced CD8^+^ T cell subset responses, skewing of T cell clonality and greater proportional repertoire occupancy by large clones. Finally, analysis of TCGA data suggests that risk allele carriers independently have improved melanoma survival. These observations highlight key roles for B cells and IL-7 in both ICB response and toxicity and clinical outcomes in melanoma.

## Abstract (web summary)

Genetic analyses in a cohort of patients with melanoma receiving immunotherapy reveal that variants in *IL7* are associated with immune-related adverse events and highlight the role of B cells in mediating toxicity.

## Main

Immune checkpoint blockade (ICB), consisting of monoclonal antibodies targeted against the proteins PD-1 and CTLA-4, can elicit durable remission of metastatic melanoma in a subset of individuals^1–3^ but is associated with autoimmune complications (immune-related adverse events (irAEs)), for which risk factors are poorly characterized^4–6^. Both oncological outcomes and occurrence of irAEs after ICB treatment vary markedly between patients^1,7^. We postulate that irAE development can be influenced by cancer-independent factors, including germline genetics. A parallel genome-wide association study (GWAS) identified carriage of the minor allele of rs16906115, a non-coding polymorphism at *IL7*, to be associated with increased risk of ICB-associated irAEs. In the present study, we replicated this clinical association in a cohort of 214 patients receiving ICB for melanoma, finding that carriers of the minor allele of rs16906115 (referred to as the risk allele) have significantly increased risk of toxicities requiring corticosteroid treatment. RNA sequencing (RNA-seq) performed on isolated immune cell types from patients pre-ICB and post-ICB treatment (*n* = 194), and healthy controls (*n* = 170), demonstrates markedly increased *IL7* expression in patient-derived B cells before treatment, with this effect being significantly greater in risk allele carriers. Notably, independent of genotype, we found that increased B cell *IL7* expression pre-treatment is associated with risk of irAEs, indicative of causality. By performing single-cell (sc) RNA-seq of B cells from patients and controls, we delineated B cell subsets expressing *IL7* and note that risk allele carriage is associated with increased B cell maturation. Finally, reflecting the importance of IL-7 in T cell maturation, we found genotype-specific T cell receptor (TCR) repertoire skewing with increased proportional repertoire occupancy by hyper-expanded CD8^+^ T cell clones in response to ICB treatment. Separate analysis of The Cancer Genome Atlas (TCGA) data demonstrates that the risk allele is associated with disease-specific and overall survival in melanoma, indicating that this pathway plays a role in the natural history of melanoma progression. In summary, we replicated and mechanistically dissected the effects of the leading identified risk locus for ICB-induced irAEs, implicating a key role for B cells and IL-7 in mediation of immunological responses and toxicity to these therapies.

## Results

### rs16906115 is a risk allele for irAE development post-ICB

**Fig. 1:**
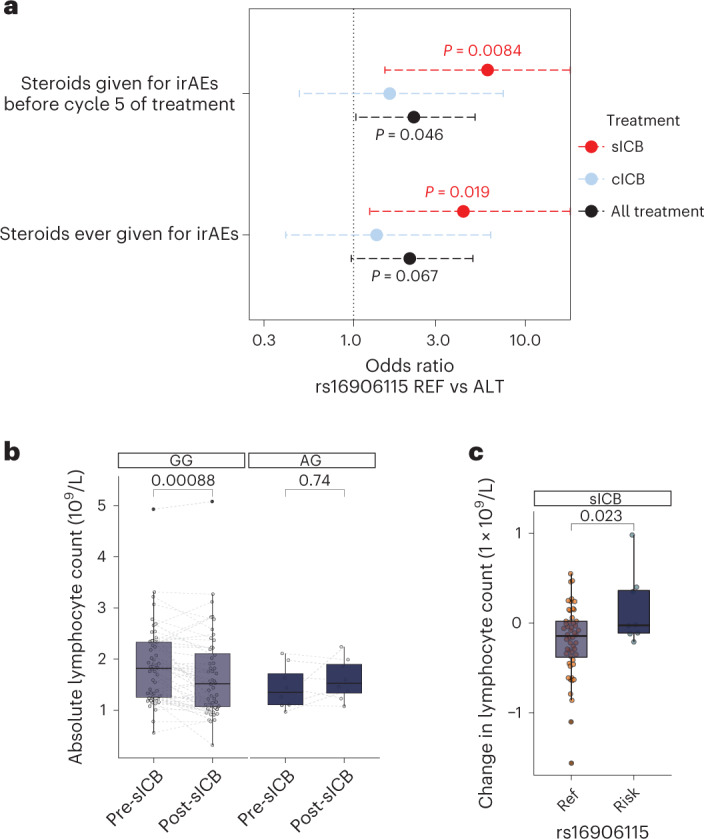
Association of rs16906115 with ICB-associated irAEs and flux in lymphocytes. **a**, OR for developing irAEs requiring steroids before C5 of treatment, or ever, according to allele carriage of rs16906115 and treatment type, and all ICB. Whiskers represent 95% CI; two-sided Fisher exact test, *n* = 214 patients (98 sICB and 116 cICB). **b**, Pre-treatment versus first post-treatment lymphocyte count for patients receiving sICB, split by allele; two-sided Wilcoxon signed-rank test, *n* = 60 patients. **c**, Difference between pre-treatment and post-sICB treatment lymphocyte count by allele; one-sided Student’s *t*-test, *n* = 60 patients.

**Extended Data Fig. 1:**
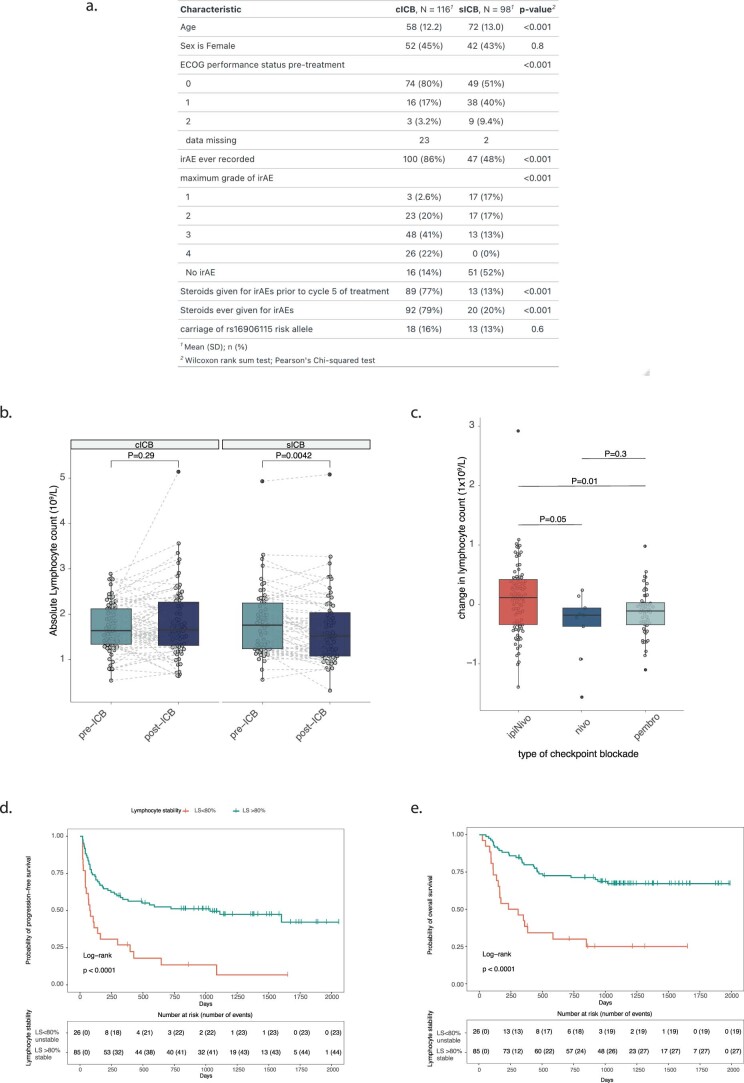
Extended Clinical Data. **(a)** Details of patient cohort, tests are as specified and were two-sided (**b**) Comparative response of lymphocyte count to cICB and sICB; two-sided Wilcoxon signed rank test, n = 134 patients (74 cICB, 60 sICB). **c)** Comparison of fall in lymphocyte count in recipients of nivolumab versus those of pembrolizumab; two-sided T tests (n = 74 ipi/nivo, n = 9 nivo, n = 51 pembrolizumab). (**d**) Kaplan Meier analysis of progression-free survival in immunotherapy naïve metastatic melanoma patients within the cohort according to LS (unstable defined as post-treatment lymphocyte count ≤80% pre-treatment value); log-rank test (n = 111). (**e)** As per (**d)**, but curves represent overall survival across cohort according to LS (n = 111). For boxplots in Extended Data Fig. 1b, c central line reflects the median, the box reflects the 25%–75% quartiles, upper whisker extends to largest value no further than 1.5x interquartile range, lower whisker extends from 25% quartile to the smallest value no further than 1.5x interquartile range.

We sought to replicate the reported association between rs16906115 and irAE development in a dataset of 214 prospectively recruited and genotyped patients who had received ICB for melanoma (Extended Data Fig. 1a), in which rs16906115 had a minor allele frequency (MAF) of 7.4%. We found that carriage of the minor risk (A) allele (one or two copies) was associated with an increased likelihood of developing severe (grade 3 or higher) irAEs requiring corticosteroids before the fifth cycle (C5) of treatment across all patients, with an odds ratio (OR) of 2.24 (95% confidence interval (CI): 1.03–5.09, *P* = 0.046) (Fig. 1a). In the primary irAE GWAS dataset, 90% of patients were treated with single-agent anti-PD-1 (sICB) or anti-PDL1, as opposed to combination anti-CTLA-4/anti-PD-1 (cICB). Given that sICB recipients have only ~20% the risk of cICB recipients for developing irAEs^1,3,7^, the sensitivity to detect genetic effects is likely greater with this more moderate treatment. In keeping with this, we found that sICB (anti-PD-1) recipients carrying the risk allele had a 6.0 OR (CI: 1.5–23.0, *P* = 0.0084) of developing irAEs requiring steroids before C5, as well as increased risk of requiring steroids at any timepoint on treatment (OR 4.4, 95% CI: 1.2–15.1, *P* = 0.019) (Fig. 1a). The single-nucleotide polymorphism (SNP) rs16906115 is intronic to *IL7*, encoding the cytokine IL-7, a key lymphopoietic cytokine with pleiotropic effects across lymphocyte subsets^8–10^. We, therefore, explored the relationship between rs16906115 status and ICB-induced changes in lymphocyte count, as measured at routine hospital blood tests. Comparison of pre-treatment lymphocyte count with the first count measured at least 21 days after treatment initiation (referred to as lymphocyte stability (LS)) revealed that, although cICB treatment did not significantly affect LS, sICB treatment elicited a fall in LS (Extended Data Fig. 1b), which was consistent across recipients of nivolumab and pembrolizumab (Extended Data Fig. 1c). In particular, non-carriers of the risk allele had significantly reduced LS post-sICB (median pre-treatment value 1.82 × 10^9^ cells per liter, interquartile range (IQR) 1.26–2.33; post-treatment value 1.52 × 10^9^ cells per liter, IQR 1.07–2.11) (*P* = 0.00044; Fig. 1b), a reduction not observed in risk allele carriers (median pre-treatment value 1.35 × 10^9^ cells per liter, IQR 1.11–2.72; post-treatment 1.53 × 10^9^ cells per liter, IQR 1.34–1.90) (*P* = 0.68; Fig. 1b). Correspondingly, rs16906115 status was associated with the magnitude of sICB-induced change in lymphocyte count (non-carriers: −0.15 × 10^9^ cells per liter; IQR −0.38 to 0.02 versus carriers: −0.025 × 10^9^ cells per liter; IQR −0.11 to 0.36) (*P* = 0.023; Fig. 1c). Notably, individuals with a fall in LS >20% had reduced progression-free and overall survival (Extended Data Fig. 1d,e), reinforcing the clinical importance of LS and demonstrating an association between rs16906115 and post-treatment lymphopoiesis, in keeping with the known properties of IL-7.

### rs16906115 regulates B cell *IL7* expression in patients with melanoma

**Fig. 2:**
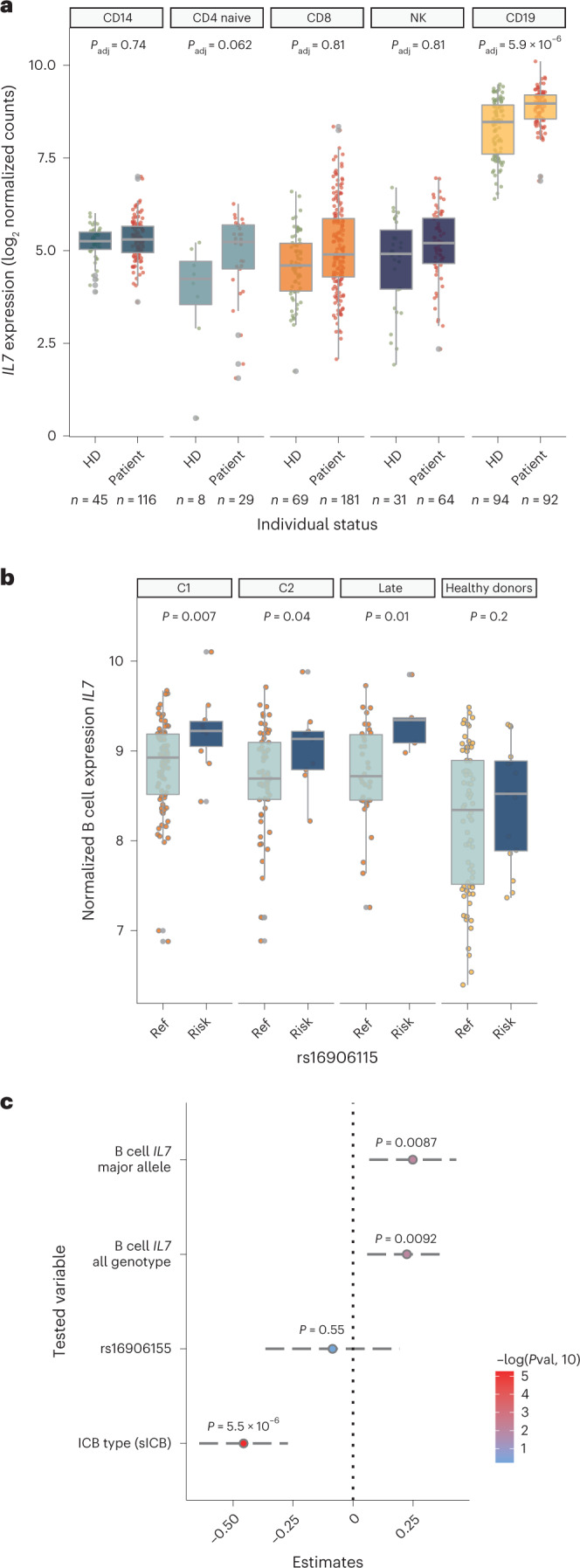
B cell IL7 expression associates with rs16906115 allele and independently with irAE risk. **a**, Expression of *IL7* measured using RNA-seq according to status (patient samples are untreated; healthy donor (HD)) split by cell type; *P* value from two-sided ANOVA, controlling for age (*n* refers to sample size for each cell type by HD or patient). **b**, Expression of *IL7* by allelic carriage and patient status; patient samples represent untreated (C1, *n* = 91), after the second cycle of treatment (C2, *n* = 68), the earliest available later sample (late, *n* = 39) or HDs (*n* = 92); one-sided Wilcoxon rank-sum test. For box plots in **a**,**b**, the central line reflects the median; the box corresponds to 25–75% quartiles; the upper whisker extends to the largest value no farther than 1.5× IQR; and the lower whisker extends from the 25% quartile to the smallest value no farther than 1.5× IQR. **c**, Results from linear mixed model random effect analysis; center point shows the estimate for the variable in association with irAE development when controlling for the other two variables; ANOVA using the Satterthwaite method; error bars mark the 95% CI; *n* = 91 patients.

**Extended Data Fig. 2:**
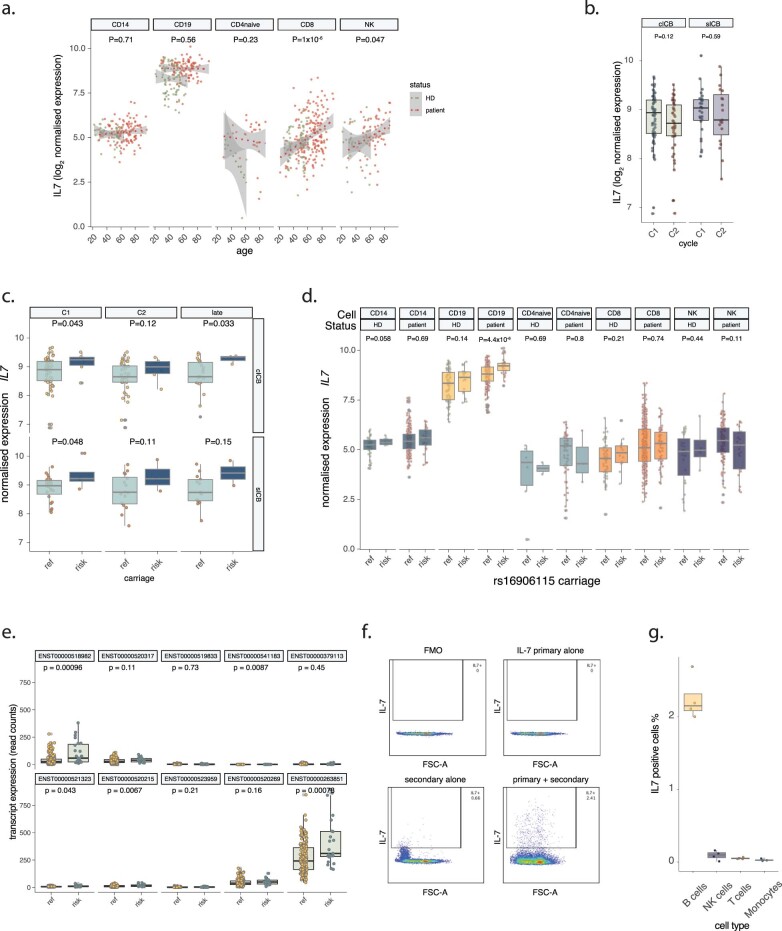
*IL7* Expression Across Cell Types. **(a)** Association between donor age and *IL7* expression across cell types, stratified by status (HD – healthy donor); P value from two-sided ANOVA, correcting for status (CD14: n = 42 HD, 216 patient samples; CD19: n = 90 HD, 159 patient samples; CD4 naïve: n = 8 HD, 50 patient samples; CD8: n = 68 HD, 326 patient samples; NK: n = 31 HD, 120 patient samples). **(b)** B cell expression of *IL7* in patient samples according to cycle and treatment type, no significant difference was noted with type or between baseline untreated and immediately prior to the second cycle of treatment (C2); two-sided T test, n = 113 cICB, n = 46 sICB. (**c)** B cell expression of *IL7* split by treatment type according to rs16906115 carriage; one-sided Wilcoxon rank-sum test, C1: n = 64 cICB patients, n = 27 sICB patients; C2: n = 49 cICB patients, n = 19 sICB patients; late: n = 24 cICB patients, n = 15 sICB patients. (**d**) Expression of *IL7* in patient and healthy donor (HD) samples dichotomized by rs16906115 status, faceted by cell type, across all samples in cohort, P value refers to result from two-sided T test between absence and presence of risk allele (one or two copies). (**e)** Expression of *IL7* transcripts annotated using StringTie^43^ according to allele status across all B cell samples showing a pan transcript effect of rs16906115 carriage; two-sided Wilcoxon rank-sum test. (**f)** Representative flow plot showing PBMCs gated on expression of CD19 with forward scatter on the X-axis versus IL-7 positive events on the y-axis. Primary = primary biotinylated anti-IL-7, secondary = Streptavidin PE, FMO = fluorescence minus one. (**g)** Analysis of samples demonstrated consistent detection of IL-7 positive B cell population in PBMCs from patients (n = 2) and controls (n = 2), with IL-7 positive fractions not detected in cells gated for CD3 (T cells), CD56+, CD3- (NK cells) or CD14 (monocytes). For boxplots in Extended Data Fig. 2b–e, g; the central line reflects the median, the box 25%–75% quartiles, upper whisker extends to largest value no further than 1.5x interquartile range, lower whisker extends from 25% quartile to the smallest value no further than 1.5x interquartile range.

Plasma IL-7 concentration forms a rate-limiting determinant of T cell expansion, being regulated, in part, by peripheral lymphocyte consumption, including activated T cells^11^. In keeping with the high levels of peripheral T cell mitosis in melanoma^12–14^, plasma IL-7 levels were undetectable by ELISAs in patient samples. We, therefore, explored *IL7* expression across immune subsets isolated from patients and healthy individuals. Although circulating immune cells are not generally recognized to produce IL-7 (refs. ^11,15–17^), we found that peripheral blood isolated CD19^+^ B cells robustly express *IL7* at levels greatly in excess of other cell subsets. Notably, *IL7* expression in pre-treatment patient-derived B cells was significantly induced compared to that in B cells from healthy controls (94 controls and 92 patients; mean log_2_ expression 8.30 FKPM versus 8.87 FKPM, *P* = 5.9 × 10^−6^), with this effect not apparent across other subsets and independent of age (Fig. 2a and Extended Data Fig. 2a). This upregulation persisted in early post-ICB treatment samples, with neither ICB type affecting B cell *IL7* expression at day 21 (Extended Data Fig. 2b). Crucially, we found that patient risk allele carriers at rs16906115 demonstrated increased B cell *IL7* induction versus non-carriers both pre-treatment and over the course of treatment, but genetic effects were not observed across other cell types in patients (Fig. 2b and Extended Data Fig. 2c). Conversely, genetic effects were not observed in B cells or other cell types in healthy individuals (Extended Data Fig. 2d), indicating that rs16906115 forms a B cell *cis* expression quantitative trait locus (eQTL) in the context of melanoma-associated immune activity. We did not identify genotype-associated B cell *IL7* splice variation but, instead, observed upregulation of the main *IL7* transcripts, indicating activity through modulation of total *IL7* gene expression (Extended Data Fig. 2e). Finally, using flow cytometry, we were able to consistently detect an IL-7^+^ population of B cells in peripheral blood mononuclear cells (PBMCs), with no IL-7^+^ cells detected in other major lineages (Extended Data Fig. 2f,g). To further assess evidence for B cell *IL7* directly mediating the risk effect of rs16906115 on irAE development, we tested association using random effects mixed linear models^18^. Consistent with B cell *IL7* mediating the genotypic association, we found that, when controlling for ICB type and rs16906115 status, pre-treatment B cell *IL7* was independently associated with irAE risk (*P* = 0.0092), whereas, when controlling for B cell *IL7* and ICB type, no genotypic effect was apparent (Fig. 2c). This remained the case when restricting analysis to individuals carrying the reference allele (*P* = 0.0087), underlying the importance of B cell *IL7* to irAE development and providing mechanistic insight into rs16906155 functionality.

### B cell *IL7* expression is associated with maturation

**Fig. 3:**
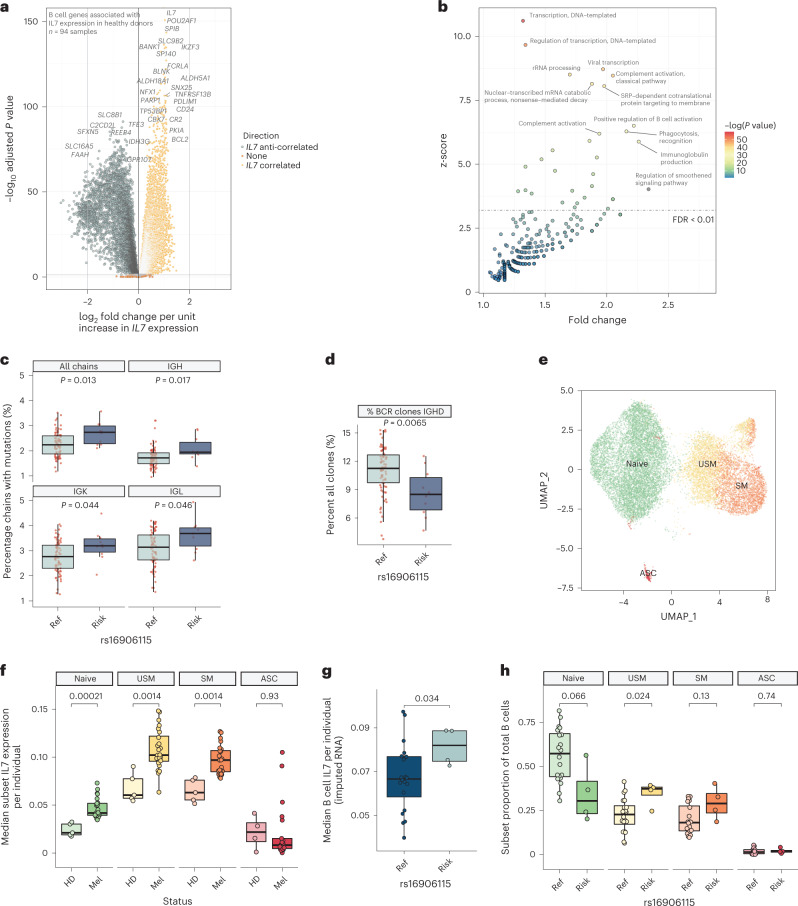
B cell *IL7* expression is associated with pleiotropic effects. **a**, Volcano plot demonstrating genes correlated with expression of *IL7* from RNA-seq data identified using *DESEQ2* from 94 healthy donor (HD) samples, two-tailed Wald test. **b**, GOBP pathway analysis of genes positively correlated with B cell *IL7* (FDR < 0.05); *x* axis demonstrates fold change above background; *y* axis demonstrates z-score; all points above dotted line FDR < 0.01; one-tailed hypergeometric test. **c**, Percentage B cell receptor chains mapped from pre-immunotherapy B cell samples with germline divergent mutations dichotomized by carriage of rs16906115 risk allele, faceted by gene; two-sided Wilcoxon rank-sum test, *n* = 91. **d**, Percentage of all B cell clones detected expressing unswitched immunoglobulin *IGHD* in pre-treatment patient samples dichotomized by carriage of rs16906115 risk allele; two-sided Wilcoxon rank-sum test, *n* = 91. **e**, UMAP of scRNA-seq B cells from *n* = 24 pre-treatment patients and *n* = 5 healthy donors (15,755 cells total), demonstrating four main populations: naive, USM, SM and ASC. **f**, Relative *IL7* expression across each B cell subset dichotomized according to HD or untreated patient (Mel); two-sided Wilcoxon rank-sum test, *n* = 24 pre-treatment patients, *n* = 5 HDs. **g**, Expression of *IL7* across all untreated patient B cells in scRNA-seq data dichotomized according to rs16906115 status; two-sided Wilcoxon rank-sum test, *n* = 24 pre-treatment patients. **h**, Frequency of B cell subsets in untreated patients from scRNA-seq data dichotomized according to rs16906115 status; two-sided Wilcoxon rank-sum test, *n* = 24 pre-treatment patients. For box plots in **c**,**d**,**f**–**h**, the central line reflects the median value; the box corresponds to 25–75% quartiles; the upper whisker extends to the largest value no farther than 1.5× IQR; and the lower whisker extends from the 25% quartile to the smallest value no farther than 1.5× IQR.

**Extended Data Fig. 3:**
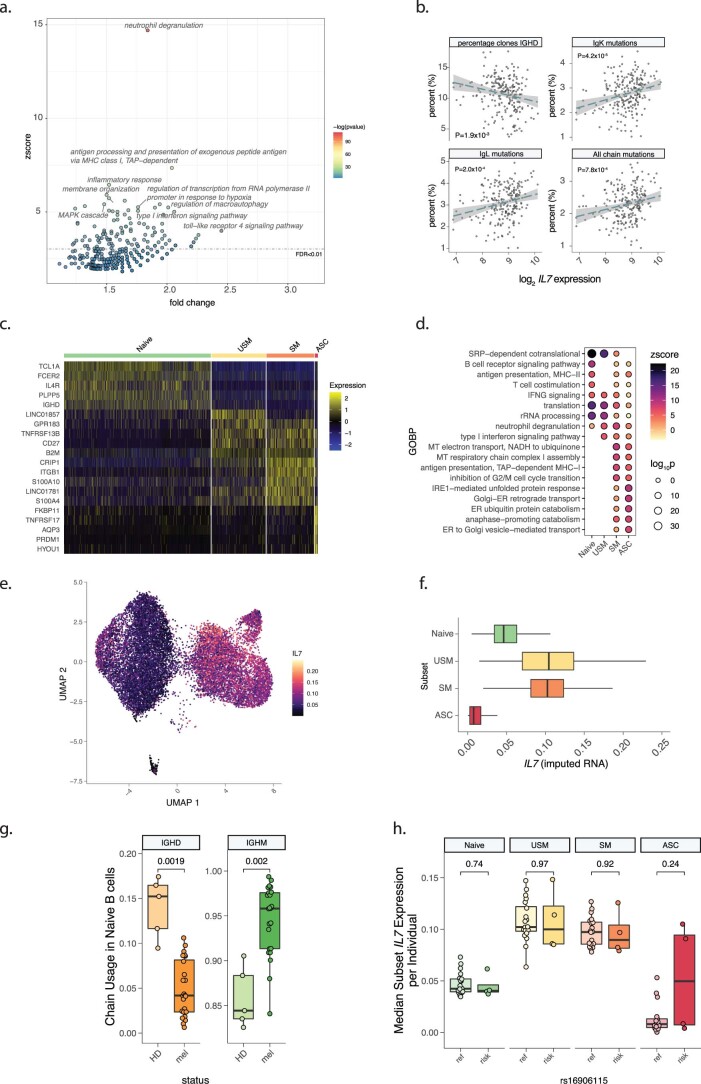
B cell correlates of *IL7* expression. (**a)** GOBP pathway analysis of genes anti-correlated with B cell *IL7* (FDR < 0.05), x -axis demonstrates fold-change above background, y-axis z score, all points above dotted line FDR < 0.01, one-tailed hypergeometric test. (**b**) Percentage chains detected with mutation from germline (chain denoted in label) or clones containing IGHD (y axis) versus relative expression *IL7* for that sample (x axis), P value refers to Spearman rank-test result, dotted lines reflect fitted linear model, shaded area 95% Confidence Interval. (**c)** Heatmap of key B cell subset specific genes from scRNA-seq data. **(d)** GOBP analysis of genes differentially expressed between B cell subsets, top 5 pathways per subset listed, one-tailed hypergeometric test. (**e)** UMAP from main Fig. 3e, depicting relative *IL7* expression per cell. (**f)** Relative expression of *IL7* (imputed) using scRNA-seq dataset across all B cells separated by subset (n = 28 samples). (**g)** Immunoglobulin chain usage in Naïve B cell subset according to patient status (HD - healthy donor n = 5, mel – metastatic melanoma n = 23); two-sided Wilcoxon rank-sum test. (**h)** Median expression of *IL7* per B cell subset from scRNA-seq data dichotomised by rs16906115 demonstrating genotype acts through altering proportions of subsets as opposed to relative expression per cell (n = 23 patients); two-sided Wilcoxon rank-sum test. For boxplots in Extended Data Fig. 3f–h the central line reflects the median value, the box corresponds to 25%–75% quartiles, the upper whisker extends to largest value no further than 1.5x interquartile range, the lower whisker extends from 25% quartile to the smallest value no further than 1.5x interquartile range.

Maturation of B cells is influenced by IL-7 (refs. ^9,10^), and so we explored the relationship between B cell development and *IL7* expression. Analysis of B cells from healthy donors demonstrated correlation of *IL7* expression with 11,305 transcripts (adjusted *P* < 0.05), including the key B cell transcription factors *IKZF3* (ref. ^19^) and *POU2AF1* (refs. ^20,21^) (Fig. 3a and Supplementary Table 2), with remarkably similar results observed in untreated patients (Supplementary Fig. 1c). Pathway analysis of B cell *IL7-*associated genes using Gene Ontology Biological Process (GOBP) datasets^22^ demonstrated enrichment in pathways associated with B cell maturation, including ‘immunoglobulin production’ and ‘positive regulation of B cell activation’, whereas anti-correlated genes were associated with TLR4 signaling, neutrophil degranulation and antigen presentation (Fig. 3b, Extended Data Fig. 3a and Supplementary Table 3). Analysis of patient-derived B cells revealed that *IL7* expression was anti-correlated with expression of the naive immunoglobulin *IGHD* but was associated with the percentage of immunoglobulin chains carrying secondary divergent mutations, as observed in somatic hypermutation (Extended Data Fig. 3b). Notably, in keeping with *IL7* expression driving these effects, we noted genotypic association of rs16906115 status, with risk allele carriers demonstrating an increased percentage of mutations in *IGH*, *IGK* and *IGL* chains in pre-treated patients (Fig. 3c). Similarly, risk allele carriers showed reduced numbers of *IGHD-*expressing B cell clones pre-treatment (Fig. 3d), indicating a pre-existent state of B cell immunoglobulin class switching and reduced B cell naivety in these individuals. To further explore *IL7* expression and genotypic association of rs16906115 across B cell subsets, we generated scRNA-seq data from B cells (*n* = 15,755 cells) from pre-treatment patients with metastatic melanoma (*n* = 24) and healthy controls (*n* = 5). We identified four major clusters of cells consisting of naive B cells, unswitched memory (USM) cells, switched memory (SM) cells and antibody secreting cells (ASCs) (Fig. 3e and Extended Data Fig. 3c,d). Expression of *IL7* was highest in the USM and SM clusters, intermediate in the naive cells and lowest in ASCs (Extended Data Fig. 3e,f) and was significantly higher in patient samples compared to healthy controls across all subsets except ASCs (Fig. 3f). Compared to those from healthy donors, patient naive B cells demonstrated reduced expression of *IGHD* and concomitant upregulation of *IGHM* (Extended Data Fig. 3g). Although only four of the patient samples carried the risk allele, we again observed increased *IL7* expression in rs16906115 risk allele carriers across all B cells (Fig. 3g), although, within subsets, there was no difference in median expression (Extended Data Fig. 3h). Instead, we found that carriage of the risk allele was associated with proportional changes in B cell subset frequency, with the eQTL effect being driven by significantly increased counts of high *IL7*-expressing USM cells and a trend to fewer naive B cells (Fig. 3h).

### rs16906115 and B cell *IL7* associate with T cell responses

**Fig. 4:**
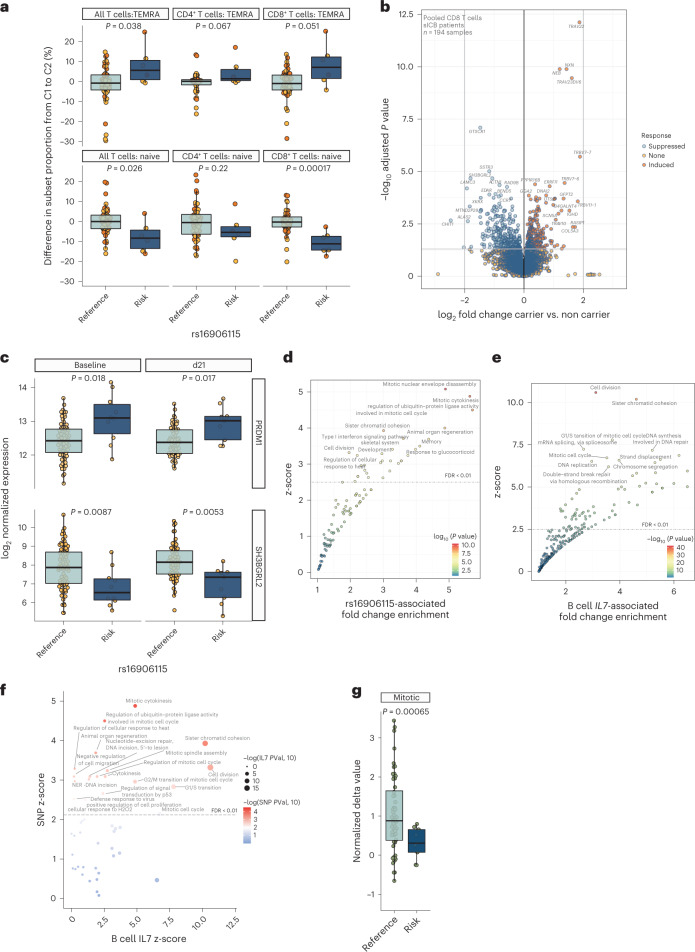
Risk allele carriage is associated with T-cell-induced ICB responses. **a**, Differences in subset proportion (depicted in facet name) between baseline untreated (C1) and immediately before the second cycle of treatment (C2) as determined by flow cytometry results assessing change in CD8^+^ T cell subset (depicted in facet name) with ICB treatment according to rs16906115 status; two-sided Wilcoxon rank-sum test, *n* = 54 patients. **b**, Volcano plot depicting results for *DESEQ2* differential gene expression analysis of CD8^+^ T cell RNA-seq data, dichotomized by rs16906115 status; *n* = 194 samples across three cycles (untreated, C2 and C4) of sICB treatment, two-tailed Wald test. **c**, Example box plots of genes regulated by allele in sICB recipients, faceted by timepoint; two-sided *t*-test of normalized expression values, *n* = 86 patients at baseline, *n* = 69 patients at day 21. **d**, GOBP pathway analysis of genes suppressed in carriers of risk allele (as depicted in **b**); one-tailed hypergeometric test. **e**, GOBP pathway analysis of genes anti-correlated in patient CD8^+^ T cells with increasing B cell *IL7* expression from the same blood samples; one-tailed hypergeometric test. **f**, Comparative analysis of z-scores (*x* axis: B cell *IL7* effect, *y* axis: rs16906115 effect) from pathway analysis in **d** and **e**. **g**, Change in CD8^+^ T cell mitotic signature score post-sICB according to rs16906115 status; two-sided *t*-test, *n* = 65 patients. For box plots in **a,c**,**g**, the central line reflects the median value; the box corresponds to the 25–75% quartiles; the upper whisker extends to the largest value no farther than 1.5× IQR; and the lower whisker extends from the 25% quartile to the smallest value no farther than 1.5× IQR.

**Extended Data Fig. 4:**
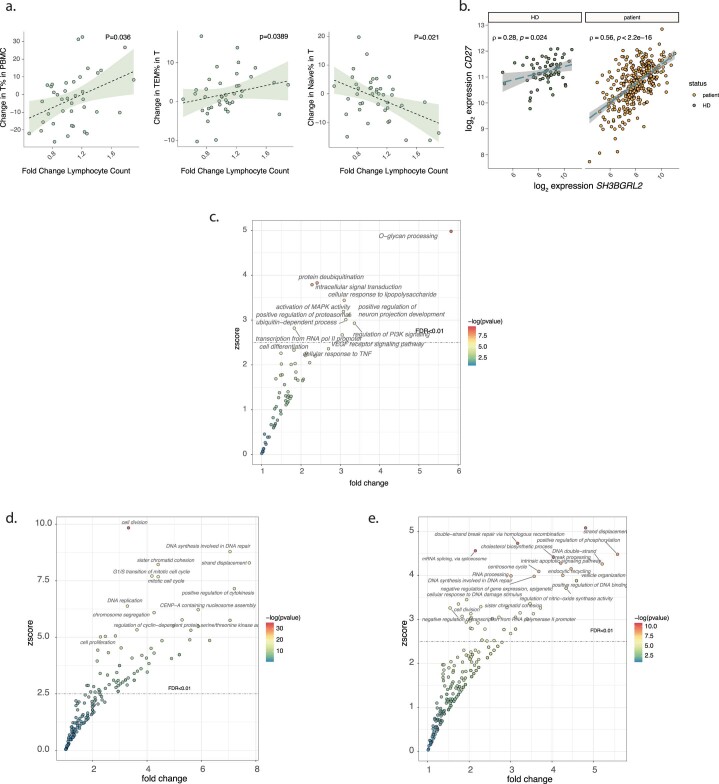
Extended T cell data. **(a)** Correlation of fold change in lymphocyte count measured on routine hospital bloods (as per Lymphocyte Stability) versus % change in cell subset as measured using flow-cytometry at the same timepoint. From left: right; % all T cells in PBMC, % T Effector Memory, % Naïve T cells; P value from Spearman rank test, dotted lines reflect fitted linear model, shaded area the 95% Confidence Interval, n = 40. (**b)** Correlation of CD8^+^ T cell expression of *SH3BGRL2* versus the naïve marker *CD27* in healthy donors (left facet) and patient samples (right facet); Spearman rank test, shaded area reflects 95% Confidence Interval of fitted linear model, P interaction with status <0.001, n = 67 controls, n = 326 patient C1/C2 samples. (**c)** GOBP pathway analysis of genes upregulated in CD8^+^ T cells in carriers of risk allele (as depicted in Fig. 4b), one-tailed hypergeometric test. (**d)** GOBP pathway analysis of genes anti-correlated in patient CD8^+^ T cells with increasing B cell *IL7* expression from same blood samples, having corrected for rs16906115 status, one-tailed hypergeometric test. **e)** GOBP pathway analysis of genes anti-correlated in patient CD8^+^ T cells with increasing B cell *IL7* expression in non-carriers of risk allele only, one-tailed hypergeometric test.

Given that T cells are central to the ICB anti-cancer response, are implicated in irAE development and are exquisitely sensitive to IL-7 (refs. ^10,12,23,24^), we explored the association between T cell ICB responses and genotype. Using flow cytometry of paired pre-treatment and post-treatment PBMC samples, carried out blinded to treatment and genotype (*n* = 54 paired samples obtained pre-treatment and immediately before the second cycle (C2), median 21 days), we observed genotypic divergence in treatment-induced subset changes. Risk allele carriers showed increases in the proportion of terminally differentiated effector memory T cells re-expressing CD45RA (TEMRA) T cells and a reciprocal fall in naive T cell proportion after ICB (Fig. 4a). In keeping with the known increased sensitivity of CD8^+^ T cells to IL7 (refs. ^25,26^), we observed the genotype-specific fall in naive T cells to be most significant in the CD8^+^ subset (Fig. 4a; median fall homozygous reference allele = −0.31%; IQR −2.80% to 2.27% versus median fall in risk allele carriers = −11.2%; IQR −14.35% to −7.45%; *P* = 0.00017). Furthermore, we explored the relationship between LS and cell subsets in these individuals, finding that LS was correlated with an increase in all T cells and effector memory cells post-ICB and a fall in naive T cells (Extended Data Fig. 4a). We proceeded to explore the effect of rs16906115 on CD8^+^ T cell gene expression across recipients of ICB. Given the stronger association of genotype with irAEs in sICB recipients and the markedly larger impact of cICB on CD8^+^ T cell transcriptomics^12^, which may saturate any genotypic effects, we restricted analysis to samples from sICB recipients. We performed differential expression analysis of CD8^+^ cell expression profiles obtained from pre-treatment and post-treatment samples from patients (194 samples from 92 patients: 86 pre-treatment and 108 post-treatment), controlling for confounding variables and dichotomizing by carriage of risk allele at rs16906115. We found that genotype was associated with 570 differentially expressed genes (DEGs) (adjusted *P* < 0.05; Fig. 4b and Supplementary Table 4), with 96% of transcripts displaying the same direction of effect of genotype across each of the three timepoints (days 0, 21 and 63), showing consistent genotypic effects over time. Supporting the flow cytometry observations, we noted genotypic associations with genes associated with divergent stages of T cell differentiation, including *PRDM1*, implicated in terminal differentiation of CD8^+^ T cells^27^. This gene was upregulated in risk allele carriers, whereas these individuals simultaneously had reduced expression of *SH3BGRL2*, which was found to strongly correlate with the naive marker CD27, most significantly in patient samples (*P*_interaction.status_ < 0.001) (Fig. 4c and Extended Data Fig. 4b). Gene pathway analysis showed that induced genes were enriched across ten pathways (adjusted *P* < 0.05), broadly encompassing anabolic processes, including ‘regulation of phosphatidylinositol 3-kinase signaling’, ‘activation of MAPK activity’ and ‘transcription from RNA polymerase II promoter’ (Extended Data Fig. 4c). Pathway enrichment for risk-allele-associated repressed genes was more marked, however, incorporating 40 pathways predominantly composed of cell division processes, including ‘mitotic nuclear envelope disassembly’ and ‘mitotic cytokinesis’ (Fig. 4d and Supplementary Table 5). To assess the evidence for B cell *IL7* expression mediating the genotypic association of rs16906115 with CD8^+^ T cell expression, we correlated CD8^+^ T cell gene expression with B cell *IL7* expression from the same blood sample (*n* = 189 sICB samples). This identified 935 significant DEGs (adjusted *P* < 0.05; Supplementary Table 6) associated with B cell *IL7*. Pathway analysis of anti-correlated genes demonstrated a striking enrichment of those involved in cell division (Fig. 4e and Supplementary Table 7), overlapping highly with pathways repressed by carriage of rs16906115 risk allele (Fig. 4f) and, thus, providing further support for mediation of rs16906115 genotypic effects via B cell *IL7* expression. In keeping with this, when controlling for rs16906115 status, B cell *IL7-*repressed pathways remained highly associated with cell division (Extended Data Fig. 4d and Supplementary Table 8). Finally, we performed the same analysis in non-carriers of the risk allele. This approach reduced the size of the cohort and the range of B cell *IL7* expression, resulting in fewer DEGs; however, at a false discovery rate (FDR) threshold of 0.2, the pathways repressed strongly reflected those in the full dataset (Extended Data Fig. 4e and Supplementary Table 9). As a further corroboration, we examined allelic associations with previously described mitotic signature scores^23^, exploring the change in signature upon treatment across each allele. Consistent with our pathway analysis results, we note significantly lower induction of the mitotic signature in risk allele carriers (Fig. 4g).

### rs16906115 associates with post-ICB CD8^+^ T cell clonality

**Fig. 5:**
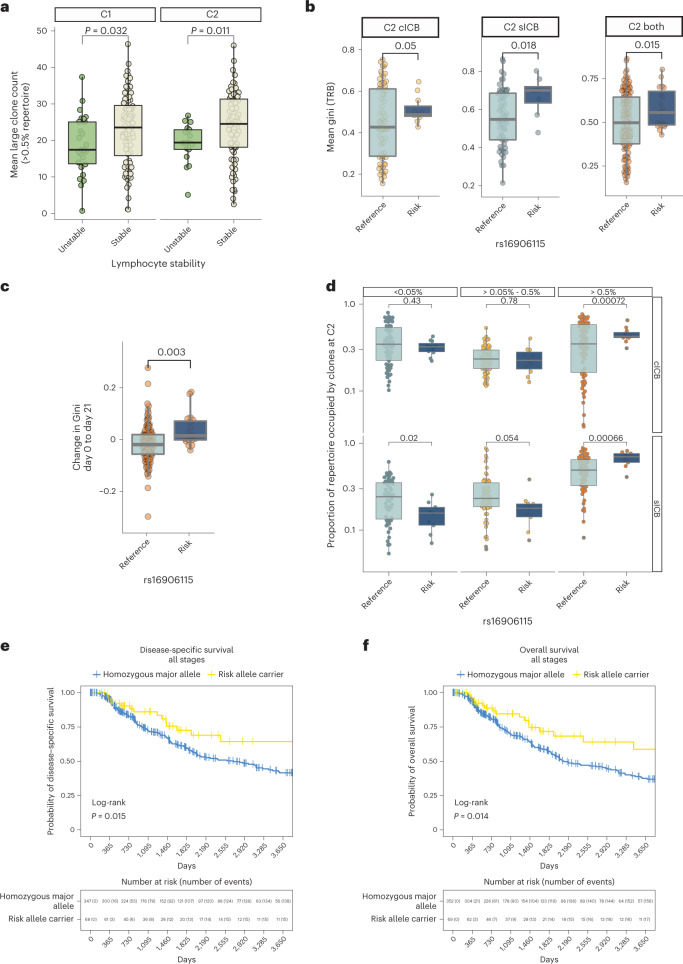
Risk allele is associated with CD8 clonality and independently associates with survival in TCGA data. **a**, Association between count of large CD8^+^ T cell clones (defined as those >0.5% repertoire in size using *TRB*) and LS for same individual pre-ICB (C1, left facet) and immediately before the second cycle of treatment (C2, right facet); two-sided *t*-test, *n* = 110 patients (C1), *n* = 91 patients (C2). **b**, Gini index (measured on *TRB* chain) immediately before C2 samples from CD8^+^ T cells across all ICB treatments with available measurement, stratified by carriage at rs16906115; two-sided *t*-test, *n* = 62 sICB patients, *n* = 76 cICB patients, *n* = 138 both. **c**, Change in Gini index between pre-treatment (C1) and post-treatment (C2) by ICB type; two-sided *t*-test, *n* = 133. **d**, Proportion of post-treatment clones per repertoire at C2 defined by *TRB* CDR3 sequence and V and J gene usage at different size thresholds (left to right facets) and by ICB type (cICB upper, sICB lower), dichotomized by rs16906115 carriage; two-way *t*-test, *n* = 77 patients cICB, *n* = 69 patients sICB. For box plots in **a**–**d**, the central line reflects the median; the box corresponds to 25–75% quartiles; the upper whisker extends to the largest value no farther than 1.5× IQR; and the lower whisker extends from the 25% quartile to the smallest value no farther than 1.5× IQR. **e**, Carriage of rs16906115 minor allele is associated with disease-specific survival in TCGA melanoma dataset; log-rank test. **f**, Carriage of rs16906115 minor allele is associated with overall survival in TCGA melanoma dataset; log-rank test.

IL-7 is crucial for the survival and proliferation of mature T cell clones^28^, and we previously observed that clonal CD8^+^ T cell proportions, both pre-treatment and post-treatment, are prognostic for long-term clinical outcome^12,23^. To assess whether rs16906115 impacts overall clonal features, we analyzed CD8^+^ T cell clone sizes, based on unique TCR CDR3 nucleotide sequences, across the cohort (*n* = 146 CD8^+^ T cell samples taken immediately before C2, of which 133 had paired baseline samples), blinded to expression phenotype. We first examined the relationship between LS and clone size, and we found that patients with stable LS (increase in count or fall <20%) had significantly more large clones (defined as those comprising >0.5% of the TCR repertoire) both pre-treatment and post-treatment (Fig. 5a), in keeping with both parameters being positively associated with beneficial response to ICB. We subsequently explored the effect of rs16906115 genotype on the Gini index of the TRB chains, a measure of clonal unevenness^29^, across pre-treatment and post-treatment samples. Notably, we found a significant interaction between ICB treatment and genotype in the change in Gini in response to treatment, with risk allele carriers showing increased clonal inequality post-ICB (Fig. 5b), resultant in skewed clonality after ICB treatment according to allele (Fig. 5c). Finally, we explored the relationship between clonal composition post-treatment according to genotype, and we found that risk allele carriers post-sICB had a repertoire composed of proportionally fewer small clones (<0.05% repertoire in size) and, instead, demonstrated significantly greater repertoire occupancy by large clones (>0.5% repertoire in size) after both cICB and sICB (Fig. 5d).

### rs16906115 is associated with melanoma survival in TCGA

**Extended Data Fig. 5:**
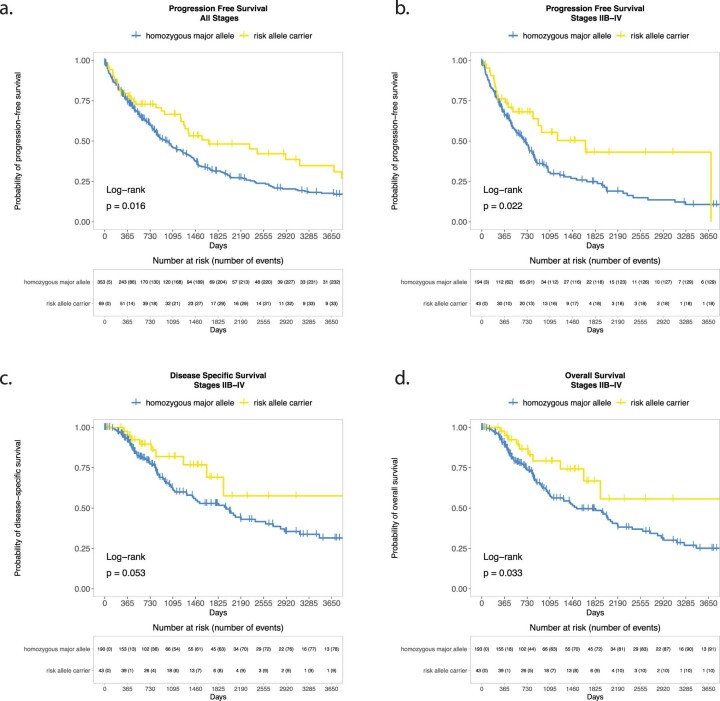
TCGA survival data by rs16906115 carriage. **(a)** Progression-free survival according to rs16906115 allele status across All Stages in TCGA melanoma dataset. (**b)** As per Extended Data Fig. 5a, but Stages IIB-IV. (**c)** Carriage of rs16906115 minor allele is associated with disease-specific survival in Stages IIB-IV in TCGA melanoma dataset. (**d)** Overall Survival according to rs16906115 allele status across Stages IIB-IV Stages in TCGA melanoma dataset. All P values correspond to log-rank test.

Given that rs16906115 is associated with baseline pre-treatment differences in patient B cell *IL7* expression and B cell maturation, we sought to explore whether this SNP had a relationship with the natural history of melanoma. We examined the frequency of this allele in the TCGA melanoma data series, and we found that it did not differ to that in our cohort (8.3% versus 7.5%, *P* = 0.72, chi-squared test). We then examined the clinical outcome data from this cohort^30^, restricting analysis to those whose ethnicity is recorded as ‘white’ and dichotomizing outcomes according to allele carriage (*n* = 435). Strikingly, we found that carriage of the risk allele was associated with increased progression-free survival (*P* = 0.016, log-rank test), disease-specific survival (*P* = 0.015) and overall survival (*P* = 0.014) (Extended Data Fig. 5a and Fig. 5e,f). This provides further independent evidence as to the importance of this allele in melanoma, inferring that, in addition to predisposing patients to ICB toxicity, B cell *IL7* plays a role in the natural history of melanoma.

## Discussion

Allelic variation at rs16906115 is a significant risk factor for development of irAEs, most notably in recipients of sICB who have a greatly reduced risk of severe irAEs. Our data replicate the co-reported GWAS by Groha et al. and provide novel insights into mechanisms of response to ICB and irAE risk. The allele rs16906115 is intronic to *IL7*, and, unexpectedly, we found robust expression of *IL7* in B cells, which is markedly induced in patients with melanoma. Notably, patient carriers of the rs16906115 risk allele demonstrate significantly greater induction of B-cell-specific *IL7*, a context-specific effect not observed in those without melanoma or in other immune subsets^31–33^. Using scRNA-seq, we replicated both patient-specific induction and genotypic association, providing further clarity regarding *IL7* expression across B cell subsets and demonstrating altered frequency of these according to rs16906115 status. Notably, supporting the effect of rs16906115 being mediated via B cell *IL7*, we demonstrate that B cell *IL7* is more significantly associated with irAE risk than rs16906115 genotype, with genotypic effect being completely resolved by controlling for B cell *IL7*. Although we did not observe rs16906115 effects on other circulating immune cells, given that stromal and epithelial cells are key sources of IL-7 (refs. ^11,15^) and considering the important role of tertiary lymphoid structures in immune responses after ICB^34,35^, we postulate that other B cell-containing lymphoid tissues may show similar context-specific *IL7* expression linked to this allele. We found that rs16906115 is associated with increased LS post-sICB and that LS anti-correlates with induction of naive T cells. Instead, carriage of the risk allele shifts the CD8^+^ T cell response to ICB from a hyper-proliferative state with expansion of naive subsets to one with reduced overall mitosis and maintenance of differentiated effector subsets, in keeping with known properties of IL-7 and previous observations regarding T cell mitosis and plasma IL-7 (refs. ^14,28,36^). This is also seen in terms of CD8^+^ T cell gene pathways differentially expressed in the presence of the risk allele and the regulation of a signature gene set that acts as a proxy for ICB-induced mitosis. Crucially, again in keeping with the effects of rs16906115 on CD8^+^ T cells being mediated via B cell *IL7*, we note that the same pathways are repressed by increasing expression of B cell *IL7* when correcting for rs16906115, with this association being present in individuals homozygous for the reference allele. Finally, analysis of CD8^+^ TCR, agnostic to gene expression profiles, corroborates the mechanistic consequences of rs16906115 allelic variation in melanoma, with patients who carry the risk allele displaying distinct clonal responses to treatment, consisting of clonal skewing characterized by fewer small clones but increased repertoire occupancy by expanded large clones. Although IL-7 has been described as an important mediator of naive T cell homeostasis in the resting state, it also has a vital role in T cell activation and memory formation^10,25,26,37–39^. Consequently, these observations suggest the importance of the latter in the context of malignancy-related chronic antigen stimulation and ICB therapy. Although the risk-allele-associated increases in large CD8^+^ T cell clone populations would be anticipated to provide a more cytotoxic response to both tumor and other tissues, we cannot discount a direct B cell-mediated role for increased irAE risk associated with this allele, supported by increased class switching and immunoglobulin chain mutations in carriers pre-treatment. The large effect size of this polymorphism reflects the central role of IL-7 in both B and T cell lymphopoiesis. Future studies are required to ascertain whether induction of peripheral B cell *IL7* is observed in other malignancies, potentially forming a biomarker for immune sensitivity. The finding of improved disease-specific and overall survival in patients with melanoma carrying the risk allele in the TCGA dataset indicates that IL-7 plays a role in the natural history of melanoma and further highlights the potential therapeutic importance of this pathway. This study highlights the power of agnostic genetic analyses to provide insights into human immunity of high relevance to disease and delineates a key role for IL-7 in response to ICB, revitalizing previous proposals for incorporating this molecule as a potential adjunct to immunotherapy strategies^36,40,41^.

## Methods

### Participants

Adult patients referred to receive ICB as standard-of-care therapy for the treatment of metastatic melanoma or in the adjuvant setting at Oxford University Hospitals NHS Foundation Trust were prospectively and consecutively approached for recruitment. All patients provided written informed consent to donate samples to the Oxford Radcliffe Biobank (Oxford Centre for Histopathology Research ethical approval reference 19/SC/0173, project nos. 16/A019, 18/A064 and 19/A114) and grant access to their routine clinical data; there was no compensation for this. Patients were recruited between 23 November 2015 and 30 September 2021 and received either cICB (ipilimumab 3 mg kg^−1^ plus nivolumab 1 mg kg^−1^ three times a week for ≤4 treatment cycles, followed by maintenance nivolumab) or sICB consisting of nivolumab 480 mg monthly, pembrolizumab 2 mg kg^−1^ three times a week or pembrolizumab 4 mg kg^−1^ six times a week. Patient characteristics are given in Extended Data Fig. 1a. Healthy donor participants were recruited via the Oxford Biobank (www.oxfordbiobank.org.uk; ethical approval reference 06/Q1605/55), with written informed consent. All donors were of European ancestry; 104 were female, and 66 were male, aged between 21 years and 66 years (median, 46.5 years; IQR 18 years).

### Clinical outcomes

Patient demographic and clinical characteristics were collected from the electronic patient record (EPR). Sex was determined on the self-reported assignment extracted from the EPR. irAEs were reported according to the National Cancer Instituteʼs Common Terminology Criteria for Adverse Events version 4.03. Steroids were defined as a prescription being issued for oral or intravenous corticosteroids to treat irAE(s) according to clinical indication. ‘Prior to cycle 5’ was defined as an irAE occurring before the patient had received a cumulative five doses of ICB and is a metric that has been used previously^7^. Response data were obtained from the EPR, with progression being defined either clinically or using radiological assessment according to irRECIST.1.1, performed approximately 12 weeks and 24 weeks after initiation of treatment. All patients who had received a minimum of one cycle of ICB were incorporated into the analysis irrespective of clinical outcome.

### Sample collection

In total, 30–50 ml of blood was collected into EDTA tubes (BD Vacutainer system) collected immediately before administration of ICB. PBMCs and plasma were immediately obtained from whole blood by density centrifugation (Ficoll Paque). All cell subset isolation for RNA-seq was carried out by magnetic separation (Miltenyi Biotec) using CD8, CD19 and CD14 positive selection for CD8^+^ T cells, B cells and monocytes and negative selection for natural killer (NK) cells and CD4^+^ naive cells according to the manufacturer’s instructions, with all steps performed either at 4 °C or on ice. Laboratory blood counts were obtained from the patients’ EPR with pre-treatment samples being defined as those taken closest to the first cycle of ICB, but no more than 30 days beforehand, and post-treatment samples being defined as the first sample taken at least 21 days after ICB, but no later than 49 days. Lymphocyte counts were generated using Sysmex XN series analyzers (Sysmex Corporation) in an NHS clinical diagnostic laboratory.

### RNA and DNA extraction

After selection, cells were pelleted and resuspended in 350 μl of RLT Plus buffer with 1% beta-mercaptoethanol or DTT. Samples were snap-frozen at −80 °C for batched RNA extraction. Homogenization of the sample was carried out using a QIAshredder (Qiagen), followed by RNA and gDNA extraction using the AllPrep DNA/RNA/miRNA Kit (Qiagen). RNA and gDNA were eluted into 34 μl or 54 μl of RNase-free water, respectively, and concentration was quantified by Qubit and stored at −80 °C until further analysis.

### Genotyping

Genotyping was performed on the Illumina Global Screening Array 24 version 3 (Illumina) and was available from 214 patient participants ≥18 years of age, with sufficient follow-up, who had been diagnosed with metastatic melanoma (*n* = 203) or started on adjuvant pembrolizumab or nivolumab after recent (<12 weeks) resection of metastatic lymph node or other site disease (*n* = 11) who had received ≥1 cycles of ICB as a standard-of-care therapy. Genotypes were aligned to b37, filtered for call frequency >0.9, cluster separation >0.3, SNP missingness <3%, MAF >1% and Hardy–Weinberg equilibrium (HWE) *P* > 10^−20^, before imputation using the Michigan Imputation Server version 1.2.4 with Haplotype Reference Consortium version 1 as a reference panel, using Eagle2 and Minimac4 for phasing and imputation. After imputation, SNPs were filtered for MAF >4%, HWE *P* > 10^−10^ and *r*^2^ > 0.7 and lifted-over to b38.

### Bulk RNA-seq and analysis

Bulk RNA-seq was performed on CD19^+^ B cell and CD8^+^ T cell samples derived from healthy individuals and patients receiving single or combination ICB. RNA was thawed on ice before mRNA isolation using NEBNext Poly(A) mRNA Magnetic Isolation Module Kits. Up to 600 ng of RNA was then used to generate dsDNA libraries using NEBNext Ultra II Directional RNA Library Prep Kits for Illumina (cohort sizes given in the text) as previously described^12^. Samples were then sequenced on either an Illumina HiSeq 4000 (75-bp paired-end) or a NovaSeq 6000 (150-bp paired-end) at the Oxford Genomics Centre. Differential gene expression and pathway analysis were performed using DESeq2 (ref. ^42^) version 1.30.1 controlling for batch effects, RNA concentration, age and, where multiple cycles of treatment were incorporated, cycle of treatment. No difference was noted between adjuvant patients and metastatic patients in terms of B cell *IL7* expression at baseline (*P* > 0.2) either pre-treatment or post-treatment. Transcript assembly was performed using StringTie^43^ using Ensembl GRCh37 as the reference (ftp://ftp.ensembl.org/pub/release-75//gtf/Homo_sapiens.GRCh37.75.gtf.gz) for each sample individually, before merging of assemblies and quantification of isoform expression. Comparative analysis per allele was based on read counts using a Wilcoxon signed-rank test. Mitotic signature score was calculated using DESeq2-normalized expression data, taking the geometric mean of the top 50 genes most associated with the mitotic subset in previous CD8^+^ T cell single-cell sequencing data^23^.

### Adaptive receptor analysis

B cell receptor (BCR) and TCR analysis was performed using the MiXCR^44^ version 3.0.13 package with settings as previously described^12,23^. Clonal metrics were calculated from TCR and BCR data by taking the minimum number of reads/chains recorded across samples and re-sampling other samples to the same depth, bootstrapping 1,000 times. BCR usage was generated for IGHC genes. Usage was calculated as either the percentage of IGH clones or reads expressing each individual IGHC gene segment. Mutation rate was inferred in BCR samples, representative of junctional diversity and somatic hypermutation. Information regarding base substitutions, insertions and deletions was extracted from MiXCR output files from ‘allVAlignments’, ‘allDAlignments’ and ‘allJAlignments’ columns. The number of mutations per BCR clone was calculated as the number of substitutions, insertions and deletions combined. Average mutation percentage was then calculated per BCR sample as the total number of mutations detected per clone divided by the total BCR length. Analysis was repeated by chain.

### scRNA-seq

5′ scRNA and VDJ sequencing (10x Genomics) was performed on PBMCs (kit version 2) according to the manufacturer’s instructions. In brief, cryopreserved PBMCs were thawed and purified using the Dead Cell Removal Kit. Cells from 5–8 donors were pooled, and an estimated 50,000–60,000 cells were loaded per channel. Gene expression and VDJ libraries were 150-bp paired-end sequenced on a NovaSeq 6000 (Illumina) with a targeted read depth of 25,000 reads per cell. Reads were mapped using cellranger version 6.0.1, with the union of cells called by cellranger and EmptyDroplets^45^ used downstream. Donor identification was performed using cellsnp-lite and vireoSNP^46^, and ambient RNA contamination was removed with SoupX^47^. Cells identified as genotypic doublets, and those with nFeatures <300 or mitochondrial reads >20%, were excluded. The remaining PBMCs were separately clustered and projected into two-dimensional (2D) uniform manifold approximation and projection (UMAP) space in Seurat version 4.1 (ref. ^48^), using Harmony^49^ for pool harmonization. After quality control (including removal of doublets and exclusion of TCR or dual-BCR carrying cells), clusters identified as B cells based on marker expression (that is, *CD79A* and *MS4A1*) and subset inference using SingleR^50^ were retained for further analysis. Subset clusters were annotated based on a combination of gene expression, SingleR inferences and VDJ usage, and *IL7* expression was imputed using MAGIC^51^.

### Flow cytometry

Patient PBMCs frozen in 90% FCS + 10% DMSO were thawed at 37 °C and washed once in HBSS before staining of 1 × 10^6^ cells with either LIVE/DEAD Fixable Near-IR (Invitrogen) or Zombie Green (BioLegend). Cells were washed and subsequently stained with antibodies against CD3, CD4, CD8α, CD27 and CD45RA in 5% FBS + HBSS. Alternatively, cells were stained with antibodies targeted against CD19, CD3, CD56 and CD14 along with biotinylated anti-IL-7 before staining with streptavidin-PE. Fluorescence minus one (FMO) controls along with primary biotinylated anti-IL-7 and secondary streptavidin-PE alone controls were included to determine IL-7 gating per individual. After washing, cells were fixed for 10 minutes with 2% paraformaldehyde (Sigma-Aldrich), washed and resuspended in 5% FBS + HBSS before acquisition on a BD LSRII. Data were analyzed in FlowJo (Tree Star, version 10.7.1) and R (version 4.0.5). Antibody details are shown in Supplementary Table 9, and gating was as previously performed^12^. All staining steps were performed for 30 minutes and at 4 °C unless otherwise stated.

### General statistical analysis

Statistical comparisons were performed as indicated in each figure legend and conducted in R (version 4.0.5), with graphics using ggplot2 version 3.3.5. Paired tests were used for paired samples, usually using Wilcoxon signed-rank test as indicated. For unpaired samples, the Wilcoxon rank-sum test was primarily used as per the figure legends. ORs were generated using a generalized linear model (family = ‘binomial’). Random effect mixed linear models were run in lme4 version 1.1-26 using lmerTest version 3.1-3 to explore association between B cell *IL7* and irAE development, controlling for rs16906115 and ICB type. Survival analysis was performed using survival version 3.2-11. Pathway analysis was performed using XGR version 1.1.8. Adjusted *P* values are reported using Benjamini–Hochberg correction for multiple comparisons.

### Reporting summary

Further information on research design is available in the Nature Portfolio Reporting Summary linked to this article.

## Online content

Any methods, additional references, Nature Portfolio reporting summaries, source data, extended data, supplementary information, acknowledgements, peer review information; details of author contributions and competing interests; and statements of data and code availability are available at 10.1038/s41591-022-02095-5.

## Supplementary information

Supplementary Information Supplementary Figs. 1 and 2

Reporting Summary

Supplementary Table Supplementary Tables 1–9

## Data Availability

Cell-specific expression data are made freely available via EGA (EGAC00001001482) upon completion of a data access agreement that can be obtained by contacting the corresponding author.

## Code availability

All scripts used to create figures, including the minimal dataset required to recreate figures, consisting of gene expression count data matrices, flow cytometry counts, genotyping at rs16906115 and scRNA-seq data of B cells in the form of a Seurat object, are available via the Fairfax Group bitbucket (https://bitbucket.org/Fairfaxlab/).
