# Supplementary material for: IL7 genetic variation and toxicity to immune checkpoint blockade in patients with melanoma
Source: Nat Med. 2022 Dec 16;28(12):2592–600. doi: 10.1038/s41591-022-02095-5 (PMC9800275; doi:10.1038/s41591-022-02095-5)
Supplement: Supplementary file 1 — Supplementary Figs. 1 and 2 [file 41591_2022_2095_MOESM1_ESM.pdf]

nature medicine

Article

https://doi.org/10.1038/s41591-022-02095-5

IL7 genetic variation and toxicity to immune checkpoint blockade in patients with melanoma

In the format provided by the authors and unedited

Supplementary Figure 1

a.

b.

c.

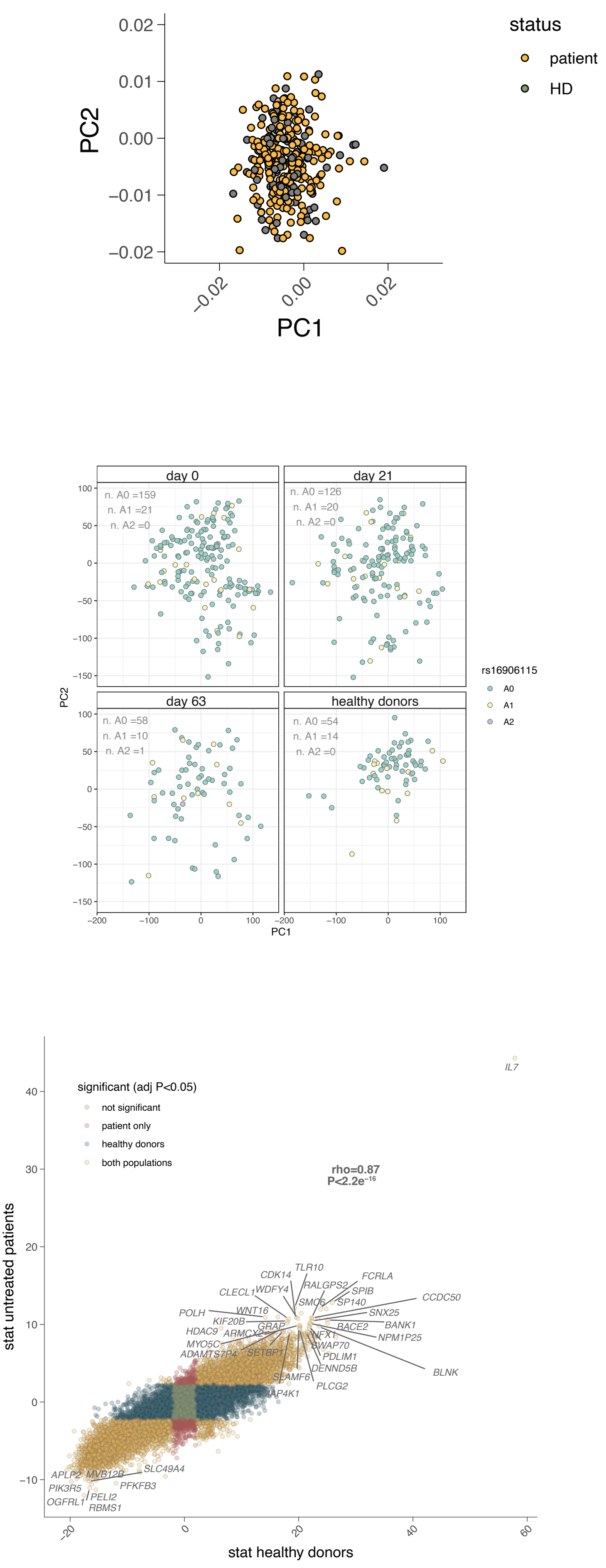

Supplementary Figure 2

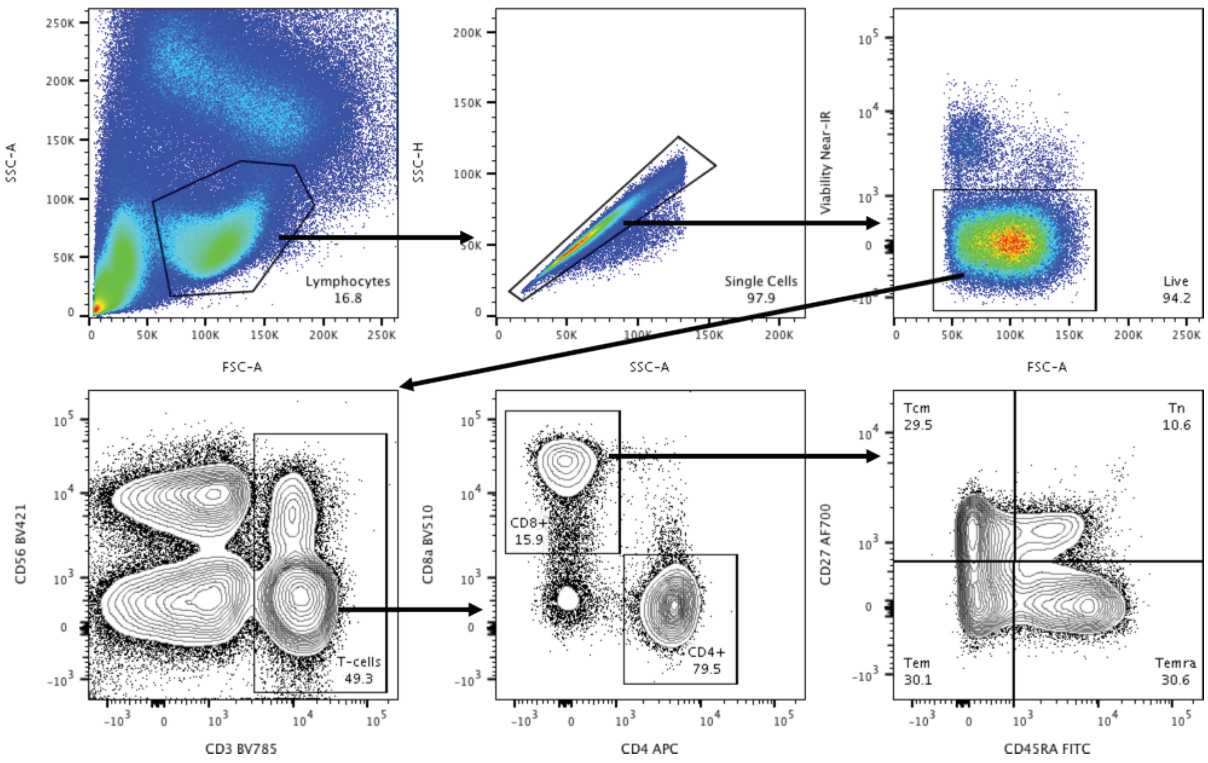

Gating strategy to identify CD8+ T-cell subsets from whole PBMCs

Antibody	Clone	Conjugate	Supplier
LIVE/DEAD™ Fixable Near-IR Dead Cell Stain Kit	n/a	n/a	Thermofisher scientific
Mouse anti-human CD3	UCHT1	FITC	Biolegend
Mouse anti-human CD56	HCD56	FITC	Biolegend
Mouse anti-human CD4	RPA-T4	APC	Biolegend
Mouse anti-human CD8a	RPA-T8	BV510	Biolegend
Mouse anti-human CD45RA	HI100	FITC	Becton Dickinson
Mouse anti-human CD27	M-T271	AF700	Biolegend
Mouse anti-human CD19	SJ25C1	BUV395	Becton Dickinson
Mouse anti-human IgD	IA6-2	BV421	Biolegend
Mouse anti-human CD16	3G8	FITC	Biolegend
