# Supplementary material for: IL7 genetic variation and toxicity to immune checkpoint blockade in patients with melanoma
Source: Nat Med. 2022 Dec 16;28(12):2592–600. doi: 10.1038/s41591-022-02095-5 (PMC9800275; doi:10.1038/s41591-022-02095-5)
Supplement: Supplementary file 2 — Reporting Summary [file 41591_2022_2095_MOESM2_ESM.pdf]

nature portfolio

Corresponding author(s): Benjamin Fairfax

Last updated by author(s): 02/10/2022

Reporting Summary

Nature Portfolio wishes to improve the reproducibility of the work that we publish. This form provides structure for consistency and transparency in reporting. For further information on Nature Portfolio policies, see our Editorial Policies and the Editorial Policy Checklist.

Statistics

For all statistical analyses, confirm that the following items are present in the figure legend, table legend, main text, or Methods section.

n/a	Confirmed	
☐	☒	The exact sample size (*n*) for each experimental group/condition, given as a discrete number and unit of measurement
☐	☒	A statement on whether measurements were taken from distinct samples or whether the same sample was measured repeatedly
☐	☒	The statistical test(s) used AND whether they are one- or two-sided<br>*Only common tests should be described solely by name; describe more complex techniques in the Methods section.*
☐	☒	A description of all covariates tested
☐	☒	A description of any assumptions or corrections, such as tests of normality and adjustment for multiple comparisons
☐	☒	A full description of the statistical parameters including central tendency (e.g. means) or other basic estimates (e.g. regression coefficient) AND variation (e.g. standard deviation) or associated estimates of uncertainty (e.g. confidence intervals)
☐	☒	For null hypothesis testing, the test statistic (e.g. *F*, *t*, *r*) with confidence intervals, effect sizes, degrees of freedom and *P* value noted<br>*Give P values as exact values whenever suitable.*
☒	☐	For Bayesian analysis, information on the choice of priors and Markov chain Monte Carlo settings
☐	☒	For hierarchical and complex designs, identification of the appropriate level for tests and full reporting of outcomes
☐	☒	Estimates of effect sizes (e.g. Cohen's *d*, Pearson's *r*), indicating how they were calculated

Our web collection on statistics for biologists contains articles on many of the points above.

Software and code

Policy information about availability of computer code

Data collection	no software was used for data collection
Data analysis	R version v_4.0.5, R packages: Deseq2 v1.30.1, ggplot2 v3.5.5 , XGR v1.1.8, survival v3.2-11, Seurat v4.1. RNA sequences were mapped with HISAT and counts retrieved with HTseq-count. Single cell sequencing mapped with Cell ranger. TCR and BCR were mapped using MiXCR v1.6. All variables are listed in the methods.<br><br>All scripts used to create figures, including the minimal dataset required to recreate figures consisting of gene expression count data matrices, flow cytometry counts, genotyping at rs16906115 and scRNAseq data of B cells in the form of a Seurat object is available via the Fairfax Group bitbucket (https://bitbucket.org/Fairfaxlab/).

For manuscripts utilizing custom algorithms or software that are central to the research but not yet described in published literature, software must be made available to editors and reviewers. We strongly encourage code deposition in a community repository (e.g. GitHub). See the Nature Portfolio guidelines for submitting code & software for further information.

Data

Policy information about availability of data

All manuscripts must include a data availability statement. This statement should provide the following information, where applicable:

- Accession codes, unique identifiers, or web links for publicly available datasets
- A description of any restrictions on data availability
- For clinical datasets or third party data, please ensure that the statement adheres to our policy

All sequencing data will be made freely available to organizations and researchers to conduct research in accordance with the UK Policy Framework for Health and

Social Care Research via a data access agreement. Sequence data have been deposited at the European Genome–phenome Archive, which is hosted by the European Bioinformatics Institute and the Centre for Genomic Regulation under accession no. EGAC00001001482. The minimal dataset required to recreate figures consisting of gene expression count data matrices, flow cytometry counts, genotyping at rs16906115 and scRNAseq data of B cells in the form of a Seurat object is available via the Fairfax Group bitbucket (https://bitbucket.org/Fairfaxlab/).

Field-specific reporting

Please select the one below that is the best fit for your research. If you are not sure, read the appropriate sections before making your selection.

☒ Life sciences ☐ Behavioural & social sciences ☐ Ecological, evolutionary & environmental sciences

For a reference copy of the document with all sections, see nature.com/documents/nr-reporting-summary-flat.pdf

Life sciences study design

All studies must disclose on these points even when the disclosure is negative.

Sample size	Sample size for genetic analysis was 214 patients, sample sizes varied according to datasets available (cells with RNA sequencing). For each test performed sample size is denoted throughout. No calculations were performed to define sample size, this being determined by patient enrollment and fulfilling inclusion criteria, namely receipt of checkpoint immunotherapy for melanoma. No samples fulfilling these criteria with genotyping at rs16906115 were excluded.
Data exclusions	No data were excluded
Replication	The genetic observation is itself a replication described in a co-submitted paper. Other datasets integrating peripheral B cell and CD8 T cell expression and genomics are not publicly available to perform independent replication, although we use a variety of techniques to replicate the observations in orthogonal approaches. For the B cell bulk RNAseq, we replicate and further define genetic effects using scRNA-seq. All attempts to replicate the primary observation replicated (as shown with genetic effect at this locus per cycle of immunotherapy). All covariates used are listed in the methods.
Randomization	Not appropriate for data as there are no control arms - the study is a descriptive retrospective analysis of prospectively recruited patients.
Blinding	All primary RNA sequencing and flow cytometry analysis was performed by investigators blinded to genotype/ clinical outcome and treatment.

Reporting for specific materials, systems and methods

We require information from authors about some types of materials, experimental systems and methods used in many studies. Here, indicate whether each material, system or method listed is relevant to your study. If you are not sure if a list item applies to your research, read the appropriate section before selecting a response.

Materials & experimental systems

n/a	Involved in the study	
☐	☒	Antibodies
☒	☐	Eukaryotic cell lines
☒	☐	Palaeontology and archaeology
☒	☐	Animals and other organisms
☐	☒	Human research participants
☐	☒	Clinical data
☒	☐	Dual use research of concern

Methods

n/a	Involved in the study	
☒	☐	ChIP-seq
☐	☒	Flow cytometry
☒	☐	MRI-based neuroimaging

Antibodies

Antibodies used

Target/stain Conjugate/dye Clone Dilution Source
LIVE/DEAD™ Fixable Near IR Viability kit Near-IR n/a 1000 Thermo Fisher Scientific
Zombie Green™ Fixable Viability Kit Zombie Green™ n/a 1000 BioLegend
CD3 BV785 UCHT1 25 BioLegend
CD4 APC RPA-T4 50 BioLegend
CD8α BV510 RPA-T8 25 BioLegend
CD27 AF700 M-T271 50 BioLegend
CD45RA FITC HI100 50 BD BioSciences
CD56 BV711 NCAM16.2 50 BD Horizon
CD14 APC M5E2 25 BioLegend
CD19 BUV395 SJ25C1 50 BD Horizon
IL-7 Biotin BVD10-11C10 50 BioLegend
Streptavidin PE n/a 250 BioLegend

Validation	Standard flow panel as validated according to manufacturers, for statements see: https://www.biolegend.com/en-us/quality/quality-assurance-certificates https://regdocs.bd.com/regdocs/qcinfo

Human research participants

Policy information about studies involving human research participants

Population characteristics	The study sample contained 120 males and 94 females, with this assignment self-reported and extracted from the electronic patient record. Sex-based analyses were not performed due to lack of statistical power. Patient samples were of European ancestry between the ages of 21-96 (median age 68, IQR 55-74). Control samples were collected via the Oxford biobank (www.oxfordbiobank.org.uk) with full ethical approval (REC 06/Q1605/55) and written informed consent from healthy volunteers of European ancestry, 104 were female, 66 male (self-reported assignment), between the ages of 24-61 (median age 49.5, IQR 34-54).
Recruitment	Healthy controls had previously enrolled within the Oxford Biobank prior to conception of this study.
Ethics oversight	Local ethical approval REC 06/Q1605/55 for healthy control samples. All patients provided written informed consent to donate samples to the Oxford Radcliffe Biobank (Oxford Centre for Histopathology Research ethical approval reference 19/SC/0173, project nos. 16/A019, 18/A064, 19/A114) and grant access to their routine clinical data, there was no compensation for this.

Note that full information on the approval of the study protocol must also be provided in the manuscript.

Clinical data

Policy information about clinical studies

All manuscripts should comply with the ICMJE guidelines for publication of clinical research and a completed CONSORT checklist must be included with all submissions.

Clinical trial registration	N/A
Study protocol	All methods are recorded in the paper
Data collection	Samples were collected from patients at onset of immunotherapy (i.e. blood taken upon cannulation for treatment). Subsequent samples were taken after sequential cycles of treatment as listed (with day 21 samples corresponding to those taken immediately prior to the second cycle of immunotherapy).
Outcomes	All patient samples were obtained from patients receiving standard of care treatment for melanoma within the NHS. Progression outcome was defined clinically or using radiological assessment according to RECIST.1.1. performed approximately 12 & 24 weeks post-initiation of treatment, whilst overall survival was measured from first treatment. Toxicity was defined as per methods, recorded in clinical data which was recovered blinded to genotype or outcome.

Flow Cytometry

Plots

Confirm that:

- [x] The axis labels state the marker and fluorochrome used (e.g. CD4-FITC).
- [x] The axis scales are clearly visible. Include numbers along axes only for bottom left plot of group (a 'group' is an analysis of identical markers).
- [x] All plots are contour plots with outliers or pseudocolor plots.
- [x] A numerical value for number of cells or percentage (with statistics) is provided.

Methodology

Sample preparation	PBMCs were prepared, frozen and stored in liquid Nitrogen in 90%FBS/10% DMSO for later use in flow. Samples were thawed and staining antibodies and dye clones, dilutions and manufacturer shown in Extended figure 12. Cells were stained in HBSS containing 5% fetal calf serum on ice and in the dark for 30 minutes, then fixed in 2% paraformaldehyde. All samples included fixable amine reactive viability dye.
Instrument	LSR II
Software	FlowJo version 10, R
Cell population abundance	Assessment of cell population abundance between patients are as described in methods.

Gating strategy | Supplementary figure 2

☒ Tick this box to confirm that a figure exemplifying the gating strategy is provided in the Supplementary Information.
